# The Role of TGF-β during Pregnancy and Pregnancy Complications

**DOI:** 10.3390/ijms242316882

**Published:** 2023-11-28

**Authors:** Baohong Wen, Huixin Liao, Weilin Lin, Zhikai Li, Xiaoqing Ma, Qian Xu, Feiyuan Yu

**Affiliations:** 1 Basic Medical Experiment Teaching Center, Shantou University Medical College, Shantou 515041, China; 20bhwen@stu.edu.cn (B.W.); 20hxliao@stu.edu.cn (H.L.); 20wllin@stu.edu.cn (W.L.); 20zkli1@stu.edu.cn (Z.L.); 20xqma@stu.edu.cn (X.M.); 2 Laboratory of Molecular Pathology, Department of Pathology, Shantou University Medical College, Shantou 515041, China; 3 Department of Cell Biology and Genetics, Shantou University Medical College, Shantou 515041, China

**Keywords:** TGF-β, pregnancy, immunology

## Abstract

Transforming growth factor beta (TGF-β), a multifunctional cytokine, is one of the most important inflammatory cytokines closely related to pregnancy. It plays significant roles in hormone secretion, placental development, and embryonic growth during pregnancy. TGF-β is implicated in embryo implantation and inhibits the invasion of extraepithelial trophoblast cells. It also moderates the mother-fetus interaction by adjusting the secretion pattern of immunomodulatory factors in the placenta, consequently influencing the mother’s immune cells. The TGF-β family regulates the development of the nervous, respiratory, and cardiovascular systems by regulating gene expression. Furthermore, TGF-β has been associated with various pregnancy complications. An increase in TGF-β levels can induce the occurrences of pre-eclampsia and gestational diabetes mellitus, while a decrease can lead to recurrent miscarriage due to the interference of the immune tolerance environment. This review focuses on the role of TGF-β in embryo implantation and development, providing new insights for the clinical prevention and treatment of pregnancy complications.

## 1. Introduction

**Figure 1:**
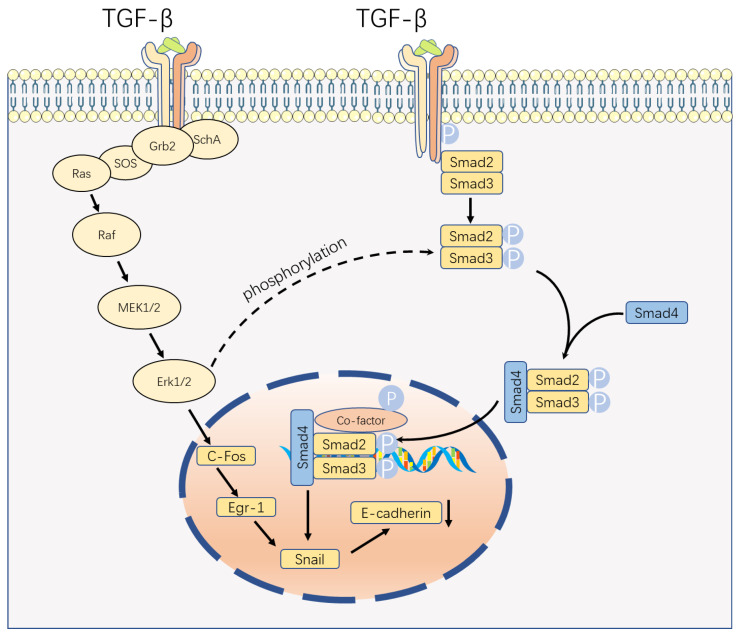
Smad-Dependent Pathway and Smad-Independent Pathways. The traditional pathway of TGF-β signaling is a Smad-dependent pathway. When TGF-β binds to the receptor, it induces the phosphorylation(p) of the Smad2/3 complex, which sequentially forms a complex with Smad4 and co-factor to mediate its biological effect. Another pathway is the extracellular signal-regulated kinase (ERK) mitogen-activated protein kinase (MAPK) pathway. Upon receptor binding, phosphorylated SchA recruits Grb2/Sos, leading to the activation of Erk1/2 through the Ras, Raf, and MEK1/2 cascade. Finally, the Erk1/2 regulates the expression of Snail, which in turn binds to the cadherin promoter and suppress its function. P, phosphorylated. Parts of the figure were drawn by using pictures from Servier Medical Art. Servier Medical Art by Servier (https://smart.servier.com, accessed on 15 May 2023) is licensed under a Creative Commons Attribution 3.0 Unported License (https://creativecommons.org/licenses/by/3.0/).

Transforming growth factor beta (TGF-β) serves as a multifunctional cytokine critical to several biological processes such as cell proliferation, differentiation, migration, and apoptosis. TGF-β includes TGF-β1, TGF-β2, and TGF-β3 [1]. TGF-β signaling can be classified into two primary pathways: the classical Smad-dependent pathway and the non-classical Smad-independent pathway (Figure 1) [2]. The basic framework of classical signal transduction pathways is highly conserved. Ligands, including TGF-β, Activins, inhibins, Nodal, and bone morphogenetic proteins (BMP), interact with transmembrane receptor type I and type II complexes on the cell surface [2,3]. This interaction leads to the phosphorylation of the GS domain (rich in glycine and serine residues) of type I receptors through constitutively active type II receptor kinases [2]. Upon phosphorylation, the GS domains recruit receptor-regulated SMADs (R-SMADs), which subsequently form complexes with the common mediator, SMAD4 [2]. Once activated, the SMAD complexes translocate to the nucleus and bind to site-specific recognition sequences in the promoter regions of target genes, directly regulating their positive and negative transcription, with the involvement of DNA-binding cofactors such as p300 and CREB-binding protein (CBP) [4].

The diversity of TGF-β signaling in cells depends not only on the various ligands, receptors, SMAD mediators, or SMAD interacting proteins but also on the ability of TGF-β to activate other signaling pathways. In the non-canonical pathway of TGF-β signaling, TGF-β receptor complexes transmit signals via other factors, such as TRAF4 or TRAF6, TAK1, p38 MAPK, RHO, PI3K-AKT, ERK, JNK, or NF-κB. This alternative signaling cascade indirectly manipulates numerous cellular processes, such as apoptosis, epithelial–mesenchymal transition, cell migration, proliferation, and differentiation, as well as extracellular matrix formation [2,3,4].

In addition to classical and non-classical routes, TGF-β signaling is also affected by other pathways, such as WNT, Hippo, Notch, interferon, TNF, and RAS pathways [5,6,7].

TGF-β1, which has multiple therapeutic effects, is a cytokine that is most prevalently and diversely expressed among the three TGF-βs and has been extensively studied. It is crucial for maintaining immunological homeostasis, sustaining immune cell functions, modulating immune cell differentiation, and promoting embryonic growth [8]. Essential processes such as cellular and tissue development, vasculogenesis, wound healing, and immunological homeostasis are all regulated by TGF-β1. Through the collaboration of different transcription factors or regulators, further layers of regulation take place after TGF-β1 binds to TGF-β receptors, ensuring the specificity of TGF-β1 signaling in a particular biological context. These intricate coordinations enable TGF-β1 to exert pleiotropic effects while maintaining high specificity [9].

TGF-β also plays a pivotal role in pregnancy. In recent years, there has been increasing interest in the relationship between TGF-β and pregnancy. The mechanism underlying TGF-β’s involvement in pregnancy is one of the current hotspots and difficulties in the field of reproductive medicine. Currently, research on the role of TGF-β in pregnancy mainly focuses on its involvement in embryo implantation, immune tolerance regulation, placental development, and its association with various pregnancy complications.

Pregnancy is a highly intricate process involving a series of physiological changes in both the mother and fetus. Numerous studies have demonstrated that TGF-β plays a crucial role in regulating these physiological changes, including critical events such as embryonic implantation, trophoblast invasion, placental development, and fetal growth. A normal pregnancy develops and is maintained in large part as a result of the carefully orchestrated invasion of trophoblast cells, the exact coordination of immune cells and cytokines, and the interaction between trophoblast and immune cells [10]. TGF-β1 is extensively involved in the regulation of immune cell function and plays an integral role in fetal–maternal immune tolerance [11]. Implantation, the key stage of developing pregnancy, is a complicated and delicately regulated process that needs molecular and cellular processes that culminate in uterine development and differentiate themselves, blastocyst adherence, invasion, and placenta creation. Furthermore, disruptions in TGF-β signaling have been associated with various pregnancy complications, including pre-eclampsia, preterm birth, and intrauterine growth restriction (IUGR).

Thus, understanding the relationship between TGF-β and pregnancy is of great significance for both basic and clinical research. From a basic research perspective, exploring TGF-β’s role in pregnancy can help us to elucidate the molecular mechanisms underlying normal and pathological pregnancies. From a clinical perspective, targeting TGF-β signaling pathways may provide new therapeutic approaches for the prevention and treatment of pregnancy-related complications. The study of the relationship between TGF-β and pregnancy has important theoretical significance and practical utility. It is helpful to understand the regulation mechanism of various physiological processes during pregnancy, to prevent pregnancy-related complications, and to develop new treatment methods.

## 2. The Effect of TGF-β on Pregnancy

TGF-β is mainly localized in the cytoplasm of villous syncytiotrophoblast and extravillous trophoblast cells during gestation. In the early stage of pregnancy, it is found within the extracellular matrix (ECM) of the villous core and decidual tissue. In the later stage of pregnancy, it appears in the cytotrophoblastic shell and decidual cells. Meanwhile, there is a decline in the concentration of TGF-β within the ECM [12,13].

### 2.1. Implantation and Placentation

#### 2.1.1. Implantation

Embryo implantation can be divided into three stages, including (1) recognizing a receptive uterine lining; (2) overlaying a blastocyst-specific signal on the receptive uterine lining before implantation; and, ultimately, (3) breaching by the developing embryo and trophoblast invasion, resulting in the growth of a placenta and decidualization [14]. The primary effect of TGF-β during pregnancy involves regulating apoptosis of decidualization cells.

Regarding the impact of TGF-β on decidualization, studies primarily focus on the TGF-β1 isoform. However, the effect of TGF-β1 in this process remains controversial. Some studies suggest that TGF-β1 promotes decidualization [15,16,17]. On the contrary, other research indicates that when human endometrial stroma cells (ESCs) are exposed to TGF-β1, they exhibit reduced levels of PRL, IGFBP-1, and tissue factor (TF) expression. This suggests that TGF-β1 may inhibit decidualization through both Smad-dependent and Smad-independent pathways [18]. In addition, TGF-β1 has been shown to suppress the expression of progesterone receptor (PR) and Dickkopf-1 (DKK, WNT inhibitor) in mature ESCs via both Smad-dependent and Smad-independent pathways, which could provide an additional route for decidualization [19].

Apoptosis plays a crucial role in the process of embryo implantation. At the site of embryo implantation, endometrial epithelial cells exhibit distinct morphological characteristics of apoptosis [20,21,22]. Evidence suggests that TGF-β may trigger apoptosis by interacting with the PI3K/Akt survival pathway and decreasing the expression of XIAP (X-linked inhibitor of the apoptosis protein) [23]. During implantation, proliferation occurs concurrently with apoptosis [24]. Numerous studies have demonstrated that TGF-β can promote the proliferation of endometrial cells in neoplastic growths, which share a similar mechanism with normal endometrium cells [25,26].

The dual role of simultaneously promoting apoptosis and proliferation is investigated in the study of Zeinab and colleagues [27]. The potential mechanisms underlying TGF-β’s dual effects include three key factors: (1) variations in the levels of different isoforms, such as TGF-β1/2, which tend to induce apoptosis in endometrial and extravillous cells, while TGF-β3 fosters proliferation; (2) the capacity of cells to adjust the types and co-expression of TGF-β receptors, thereby influencing their responsiveness to TGF-β; and (3) the presence of antagonists, such as XIAP and PPAR-γ, which can attenuate the apoptosis-inducing effects of TGF-β1/2.

#### 2.1.2. Placentation

During pregnancy, placental development is characterized by the invasion of the uterine wall and spiral arteries by extravillous cytotrophoblast (EVT) cells. These EVT cells replace the vessel wall cells, establishing a high-flow, low-resistance vascular system that ensures a steady blood supply to the placenta [28]. The invasion of EVT cells plays a crucial role in placentation. Studies have predominantly centered on TGF-β1 and its influence on EVT invasion. It has been demonstrated that TGF-β1 reduces the invasion of human EVT cells, and a similar effect is observed with TGF-β2. TGF-β1 activates the Smad2/3 pathway by phosphorylating SMAD2/3 through ALK5, which in turn upregulates the level of Snail. These transcription factors then bind to the vascular endothelial cadherin (VE-cadherin) promoter, suppressing its promoter activity [29]. However, contrasting findings show that TGF-β1-induced kisspeptin expression in human EVT cells is Smad-independent. TGF-β1 exerts its inhibitory effect by stimulating kisspeptin expression through the ERK1/2 signaling pathways [30]. Another study suggests that the function of the ERK1/2 pathway may be related to Snail, which contradicts the previous study (Figure 1) [31]. Therefore, further investigation to elucidate the role of the Smad-independent pathway in the invasion of EVT cells is necessary.

### 2.2. TGF Beta and Immune Tolerance

Maintaining a healthy pregnancy is a finely regulated process in which fetal–maternal immune tolerance plays a very important role. Immune cells and cytokines coordinate with each other and play corresponding functions to maintain fetal and maternal immune tolerance [32].

During pregnancy, the fetus carries half of the paternal genetic material, which is considered “foreign” to the maternal immune system. Therefore, a specialized protection mechanism is required to prevent maternal immune attack and ensure normal fetal development. This protective mechanism primarily relies on the functions of the placental barrier, immunomodulatory factors, and the establishment of immune tolerance. The placenta serves as a crucial interface between the mother and the developing fetus, protecting against maternal immune cell invasion through physical barriers [33]. Throughout pregnancy, both the fetus and placenta secrete various specialized immune regulatory factors such as IL-10, TGF-β [34,35], progesterone, and placental growth hormone [36]. These factors have the potential to impede maternal immune cell activity and reduce the risk of immune-mediated fetal reactions. Concurrently, pregnancy triggers a cascade of changes in the female immune system, including but not limited to the increased count of Treg cell and NK cell, which foster maternal immune tolerance towards the fetus and minimize its vulnerability to assault [37].

**Figure 2:**
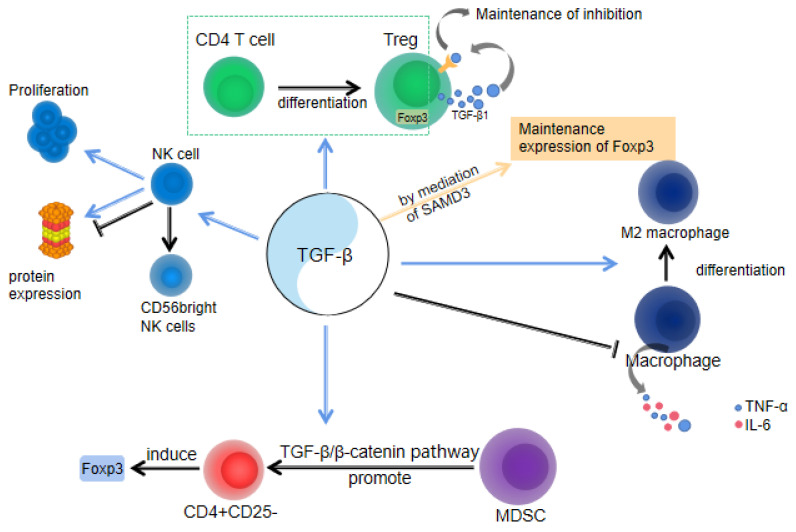
For Tregs, TGF-β1 can accelerate the transformation of the initial CD4 T cells into Tregs. TGF-β1 cells inhibit the proliferation of Th1 cells through the upregulation of cell cycle inhibitors p21 and p27 and the downregulation of CDK4-mediated cell cycle arrest in the G1 phase. TGF-β1 can promote the generation and expansion of NK cells during pregnancy. TGF-β1 also exerts a regulatory effect on NK cell protein expression, which can either positively or negatively impact the immune response mediated by these cells. M2 macrophages promote cell homeostasis, trophoblast invasion, and migration by secreting TGF-β1. Decidua G-MDSCs play an important role in promoting Foxp3 induction with CD4+CD25-T cells, and the effect is dependent on the TGFβ/β-catenin pathway.

In brief, the immune evasion mechanism during pregnancy is a complex regulatory network that facilitates normal fetal growth and development within the maternal environment, serving as a crucial safeguard for human reproduction. Simultaneously, the role of TGF-β cannot be overlooked, as it serves as an essential immunomodulator involved in various aspects of this protective mechanism (Figure 2). TGF-β is essential for the regulation of immune cells and the maintenance of immune homeostasis.

#### 2.2.1. TGF-β and Treg Cells

TGF-β signaling is important for the survival of multiple T-cell lineages [38,39,40]. Treg cells play an important role in maintaining immune homeostasis in pregnancy. Most Treg cells originate from the thymus. TGF-β is critical for the development of CD4+ CD25+ Foxp3+ regulatory T cells. The loss of TGF-β receptor I (TbetaRI) of T cells blocks the CD4+ CD25+ Foxp3+ thymocytes [41]). TGF-β is regulated by sex hormones to play an immunosuppressive role [17], which in turn affects Treg numbers in the female reproductive tract (FRT). Studies have demonstrated a close correlation between the quantities of Treg in the female reproductive tract and serum levels of estradiol [42]. In addition, male semen contains high concentrations of TGF-β [43], and exposure to semen can enhance tolerance to paternal alloantigens, suggesting that TGF-β is involved in the immunological suppression of semen [44].

#### 2.2.2. TGF-β and NK Cell

During the initial stages of pregnancy, NK cells are preferentially recruited into the endometrium to play an immunomodulatory role under the action of chemokines derived from the endometrial stroma and trophoblast cells [45].

Studies have revealed the substantial influence of TGF-β1 on the growth, maturation, and activity of NK cells throughout pregnancy. Firstly, TGF-β1 promotes the generation and expansion of NK cells during pregnancy. It exerts an inhibitory effect on the cytotoxicity of NK cells by downregulating CD16 expression on their surface, while facilitating the differentiation of NK cells into the CD56 bright subgroup with a high capacity for IFN-γ production and recognition [46,47]. These CD56 bright NK cells are widely acknowledged to play a crucial role in early gestation by safeguarding the fetus from potential harm. Additionally, TGF-β1 has a regulatory effect on NK cell protein expression, which can either positively or negatively impact the immune response mediated by these cells. For example, the combined action of TGF-β1 and IL-2 has been shown to suppress NK cell activity [46]. TGF-β1 plays a crucial role in regulating the crosstalk between NK cells and other immune and stromal cells at the maternal–fetal interface, thereby modulating placental development, fetal growth, and immune tolerance. For instance, TGF-β1 promotes the interaction of NK cells with trophoblasts, facilitating nutrient uptake by the fetus [47].

#### 2.2.3. TGF-β and Macrophages

Almost 20–30% of the white blood cells in the decidua during early pregnancy are macrophages. Macrophages play a crucial role in trophoblast invasion, vascular remodeling, and immune tolerance [48]. Research findings indicate that TGF-β exhibits potent inhibition of pro-inflammatory cytokine production, including TNF-α and IL-6, within macrophages (Figure 2). This capacity of TGF-β contributes to attenuating the body’s inflammatory response [49]. During pregnancy, both fetal and maternal metabolic processes produce a considerable amount of free radicals, which can cause oxidative damage to fetal and maternal cells. This oxidative damage is further intensified by an increased inflammatory response. Therefore, TGF-β can reduce the inflammatory response by inhibiting the production of pro-inflammatory cytokines by macrophages, thus protecting the health of the fetus and the mother. Additionally, TGF-β promotes the differentiation of macrophages into M2 macrophages [50], which possess anti-inflammatory, antioxidant, and tissue repair capabilities, promoting the healthy development of the placenta and fetus. Studies have shown that trophoblast cells induce the polarization of macrophages into the M2 subtype, which is accompanied by an increase in the expression of the M2 marker TGF-β [51]. Furthermore, TGF-β also enhances the expression of macrophage colony-stimulating factor (M-CSF) mRNA [52], as which contribute to induction of immunomodulatory processes essential for fetal development. Placental macrophage can secrete TGF-β [53], which might be an important source of TGF-β for the above immunomodulatory processes.

#### 2.2.4. TGF-β and MDSC

The myeloid suppressor cell (MDSC) is a heterogeneous population of myeloid cells and plays a key role in maintaining immune tolerance. Decidua granulocytic myeloid-derived suppressor cells (G-MDSCs) are crucial in promoting Foxp3 induction with CD4+CD25-T cells, and the effect is dependent on the TGF-β/β-catenin pathway. Research has found that the increase in G-MDSCs during pregnancy results in a decreased rate of spontaneous abortion. Compared with spontaneous abortion, G-MDSCs and TGF-β levels increased in the decidua of mice with a normal pregnancy. Additionally, G-MDSCs-induced Foxp3 induction may depend on the direct targeting of TGF-β signaling pathway [54].

In summary, TGF-β plays a multifaceted role during pregnancy by inhibiting immune cell activity, promoting immune tolerance, and maintaining immune homeostasis to safeguard fetal development and prevent maternal attack.

## 3. TGF-β and Embryonic Development

Embryonic development is a critical period of fetal differentiation and growth during pregnancy. Many cytokines are involved in the process of embryonic development, and among them, TGF-β is related to the development of numerous organ systems. The epithelial–mesenchymal transition (EMT) is considered an important event during embryonic development, forming the basis for the development of various organs. The TGF β family, including TGF-βs and bone morphogenetic protein (BMP), plays a crucial role in embryonic development as an inducer of epithelial–mesenchymal transitions (EMTs) [55,56]. EMT is characterized by the loss of endothelial features and the acquisition of mesenchymal-, fibroblast-, or stem cell-like characteristics [57,58]. Several cytokines regulate EMT, with TGF-β being the most significant [59,60,61]. The TGF-β family regulates gene expression by phosphorylating the downstream Smad signaling system through the type I and type II receptor serine/threonine kinase [62,63], thereby controlling the growth and differentiation of cells [64]. Meanwhile, some Smads such as smad6/7 can also negatively feedback TGF-β signaling [62]. The Smad signaling system contains multiple Smad-interacting proteins that collectively drive embryonic differentiation [64]. During development, TGF-β1, TGF-β2 and TGF-β3 can be respectively found in the mesoderm, early facial mesenchyme, some endodermal, ectodermal epithelial, prevertebral tissues, some mesothelial cells, and lung epithelial cells [65]. This section will describe the role of TGF-β in the development of the nervous system, respiratory system, cardiovascular system, and other organs during embryonic development.

### 3.1. TGF-β and Development of the Nervous System

The development of the nervous system during early embryonic development is a complex process, and numerous studies have demonstrated the role of TGF-β in the development of the nervous system. TGF-β regulates the initial developmental stages of the nervous system, including axon guidance, synaptogenesis, and other key events [66]. Yi et al. observed the expression of TGF-β receptors in axons during neural development and demonstrated that axons fail to form when TGF-β receptors are absent, highlighting the essential role ofTGF-β in the initiation of axon formation [67]. Luo et al. used a comparative study of dopaminergic (DA) neurons without the TGF-β type II receptor and the normal ones, both treated with TGF-β1. The results indicated that TGF-β signaling could promote the dendritic growth of DA neurons [68]. Several studies have shown that TGF-β promotes neuronal development by two processes [69], one is to prevent cell proliferation and the other is to promote neuronal differentiation. TGF-β1 prevents cells from transitioning G1 phase to the S phase by inducing the cyclin-dependent kinase (Cdk) inhibitor p21 [70] and downregulating the Cdk activator, ending cell proliferation and entering differentiation [69]. The core mechanism of neuronal differentiation depends on the classical Smad pathway for cellular regulation [69].

### 3.2. TGF-β and Development of the Respiratory System

Studies have found that TGF-β1/2/3 had different expression patterns in the respiratory system. TGF-β1 is predominantly expressed in bronchial mesoderm. TGF-β2 is exclusively expressed in endodermal bronchiolar epithelia, with increased expression observed in later stages of development. TGF-β3 exhibits varied spatial distribution at different time points: on embryonic day 12.5 (E12.5), it is mainly expressed in tracheal mesenchymal cells; on E14.5, it is predominantly expressed in endodermal epithelial cells of growing bronchioles. By E16.5, TGF-β3 is expressed in mesodermal epithelial cells [65,71]. Furthermore, it has been demonstrated that TGF-β3 plays a critical role in EMT during respiratory development. EMT is the process of epithelial-to-mesenchymal differentiation, forming the basis of lung-branching morphogenesis. Vesa et al. found that targeted inactivation of the TGF-β3 gene resulted in mice dying shortly after birth, and lung histological manifestations similar to preterm infants with bronchopulmonary dysplasia [72].

### 3.3. TGF-β and Development of the Cardiovascular System

In the vessels, TGF-β1 is involved in the development of the intima, while TGF-β2 and TGF-β3 primarily contribute to the development of the middle and adventitia of large arteries [73]. In the presence of TGF-β1, leucine-rich alpha-2-glycoprotein 1 (LRG1) promotes angiogenesis through the smad1/5/8 pathway [74]. In the heart, TGF-β1/2/3 play pivotal roles in cardiovascular development [75]. The expression of TGF-β1 is restricted to the endocardium [73]. TGF-β2 is involved in early epicardial development (E9.5–E11.5), whereas TGF-β3 is more likely to participate in later development (E11.5-onwards) [76]. BMP can also be found in the development and maturation of the epicardium [76]. Bartram et al. found cardiac and macrovascular malformations, cardiac valve malformations, and abnormalities in cardiac septation and myocardialization after the knockout of the TGF-β2 gene in mouse embryos. These malformations are similar to human cardiac malformations [77].

### 3.4. TGF-β and Development of Other Organ Systems

The TGF-β family also played an important role in the differentiation of other organ systems. Vrljicak et al. discovered that from embryonic day 12 to the end of nephrogenesis (postnatal day 15), Smads were expressed in cells and mesenchyme of the ureteric bud tip. The BMP pathway was dominant in the interstitial cells of the nephrogenic zone, while TGF-β signaling was dominant in the mesenchyme of the renal medulla [78]. While Oxburgh et al.’s study showed that Smads are downregulated after the epithelialization of mesenchymal cells [79]. Schilling and Yeh found that the TGF-β system was expressed in embryonic oocytes during the first and second trimesters of pregnancy, suggesting possible autocrine or paracrine regulatory mechanisms during ovarian development [80]. Many studies have shown that TGF-β3 is precisely expressed in medial edge epithelial (MEE) cells to promote palatal fusion [72,81,82,83], and it correlates with the timing of palatal fusion [83,84].

The development and differentiation of multiple organ systems in the human body depended on the regulation of TGF-β signaling. Consequently, it is important to investigate the pathways, roles, and influencing factors of the TGF-β family for embryonic health during pregnancy.

## 4. TGF-β in Pregnancy Complications

### 4.1. Pre-Eclampsia

Pre-eclampsia is a complication of pregnancy [16], clinically manifested by the development of hypertension, proteinuria, and systemic chronic inflammatory disease in pregnant women after 20 weeks of gestation [85]. Patients with pre-eclampsia typically exhibit decreased antioxidant capacity alongside increased placental oxidative stress [86]. Elevated levels of reactive oxygen species (ROS) can lead to increased inflammatory cytokine levels, induction of apoptosis in trophoblast cells, and elevation of blood pressure by damaging the vasodilation and contractile responses of vascular smooth muscle [87]. Yazaki et al. discovered that TGF-β1 triggered both ROS generation and Nrf2 activation in specific cells [88]. In another study, Wang et al. observed that enhanced Nrf2 expression led to reduced levels of ROS under hypoxic conditions [89]. These findings collectively imply that TGF-β1 could play a role in the development of pre-eclampsia via the ROS-Nrf2 signaling pathway. On the other hand, pre-eclampsia is associated with insufficient trophoblast cell invasion in early pregnancy [85,90], which is an excessive maternal inflammatory response to pregnancy [91]. The antigenicity of trophoblast cells is similar to that of tumors, both of which elicit immune responses, and TGF-β has been shown to act as a tumor suppressor during the initial stages of tumor growth [92,93]. This may indicate that TGF-β also inhibits trophoblast cell invasion during the first trimester of pregnancy. Lash et al. also found that TGF-β inhibits trophoblast cell invasion during the first trimester of pregnancy in their study [94]. TGF-β2 and TGF-β3 were found to be elevated in early pregnancy in patients with pre-eclampsia in some studies [95,96]. Caniggia et al. found that inhibition of TGF-β3 expression and activity in pre-eclampsia placentae restored trophoblast cell invasion [96]. These studies further confirm the role of TGF-β in inhibiting trophoblast cell invasion in pre-eclampsia patients. The precise mechanism through which TGF-β inhibits trophoblast invasion remains unclear. In the presence of TGF-β1, 2, and 3, levels of uPA and MMP9 were reduced [94], both of which can proteolytically degrade the extracellular matrix in favor of cell invasion, where uPA degrades extracellular matrix proteins by converting plasminogen to plasmin [94]. Evidence shows that TGF-β increases the invasiveness of trophoblast cells via SMAD, ERK, and MAPK14 signaling pathways [97,98], suggesting a potential therapeutic approach for addressing pre-eclampsia. Moreover, TGF-β1 has been shown to activate Nrf2 [99], and the subsequent inhibition of complete epithelial-mesenchymal transition (EMT) by Nrf2 [100] suggests that TGF-β inhibits invasion by activating Nrf2, potentially leading to insufficient trophoblast cell invasion in early pregnancy.

### 4.2. Recurrent Miscarriage

Recurrent miscarriage refers to two or more pregnancy losses occurring before 20 weeks of gestation [101]. The pathogenesis of recurrent miscarriage is related to immune disorders, which can lead to decreased maternal and fetal tolerance [102,103,104]. To the mother, the fetus is a semi-allograft with both maternal and paternal genes [105]. Under typical physiological conditions, the maternal immune system is expected to mount an immune response. However, the mother can achieve a successful pregnancy due to the establishment of a unique immune tolerance environment [103,106]. TGF-β is a cytokine that can control deleterious immune responses to the embryo [107]. Among them, TGF-β1 has immunosuppressive effects [108]. This is equivalent to creating an environment of immune tolerance. Several studies showed that TGF-β levels were lower in patients with recurrent miscarriage than in people with normal pregnancies [105,109]. It is consistent with reduced maternal–fetal tolerance in patients with recurrent miscarriage. There were several statements regarding the mechanism of reduced maternal–fetal tolerance. The study by Zhu et al. mentioned that decidual natural killer cells (dNK) are the main immune cells that maintain immune tolerance at the maternal–fetal interface [109]. Furthermore, TGF-β can inhibit the development and differentiation of human NK cells by promoting the transition of CD16+ pNK cells to CD16− cells [110]. Exogenous TGF-β can stimulate the expression of CD39 and CD73 on T cells and dendritic cells [111,112]. The latter two have been shown to convert ATP-driven pro-inflammatory immune cells into adenosine-induced anti-inflammatory immune cells and combined to inhibit the cytotoxicity of dNK cells [109,113].

### 4.3. Gestational Diabetes Mellitus

Gestational diabetes mellitus is a common complication of pregnancy, characterized by low systemic inflammation and immune system disorders [114,115,116]. TGF-β affects the activation of a variety of inflammatory cells [110,117]. At the same time, the TGF-β signaling pathway plays a key role in pancreatic development, and TGF-β/Smad3 signaling is an important regulator of insulin gene transcription and β-cell function [118,119]. Several studies have noted changes in TGF-β levels in patients with gestational diabetes [115,116,120,121,122]. TGF-β1 was significantly increased in the serum of GDM patients [121,122]. Mrizak et al. found the mRNA expression of TGF-β was increased in GDM placenta [120]. However, Yang et al. found that the reduced Treg cells in GDM patients can inhibit the pro-inflammatory response by secreting IL-10 and TGF-β, thereby reducing the risk of GDM in high-risk pregnant women [115]. These studies suggest that TGF-β plays different roles and performs multiple functions during the progression of GDM.

### 4.4. Other Pregnancy Complications

Some studies showed that low TGF-β levels increase the probability of preterm birth in women [123,124,125]. Besides, TGF-β3 was increased in people with gestational trophoblastic disease, suggesting that TGF-β3 may be significantly involved in the process of gestational trophoblastic disease [126,127].

## 5. Conclusions

TGF-β plays distinct roles in each stage of pregnancy. It exhibits various effects during embryo implantation, including the promotion or inhibition of decidualization, as well as the induction of apoptosis and proliferation to promote embryo implantation. The invasion of trophoblast cells is crucial for placental development, but TGF-β can inhibit trophoblast cell invasion, potentially leading to pre-eclampsia. Successful pregnancy necessitates maternal and fetal immune tolerance. TGF-β contributes to immune tolerance by inhibiting T cell proliferation, increasing Tregs, affecting NK cell differentiation, promoting cell homeostasis, and so on. When there is a decrease in immune tolerance, recurrent miscarriage can easily occur. Gestational diabetes mellitus, preterm birth and other pregnancy complications are also related to TGF-β. In addition, TGF-β is also crucial in the process of embryonic development, affecting the occurrence of nervous, respiratory, cardiovascular, and other systems. It is important to note that some of the conclusions discussed in this review are derived from mouse models. Although these conclusions cannot be directly generalized to humans, they provide ideas for the study of pregnancy-related diseases.
